# *Aerococcus urinae* – A potent biofilm builder in endocarditis

**DOI:** 10.1371/journal.pone.0231827

**Published:** 2020-04-23

**Authors:** Berrin Yaban, Judith Kikhney, Michele Musci, Annett Petrich, Julia Schmidt, Maria Hajduczenia, Felix Schoenrath, Volkmar Falk, Annette Moter

**Affiliations:** 1 Biofilmzentrum, Dept. of Microbiology, Infectious Disease and Immunology, Charité–Universitätsmedizin Berlin, corporate member of Freie Universität Berlin, Humboldt-Universität zu Berlin, and Berlin Institute of Health, Berlin, Germany; 2 MoKi Analytics GmbH, Marienplatz, Berlin, Germany; 3 Dept. of Congenital Heart Surgery—Pediatric Heart Surgery, German Heart Center Berlin, Berlin, Germany; 4 Dept. of Cardiothoracic and Vascular Surgery, German Heart Center Berlin, Berlin, Germany; 5 DZHK (German Centre for Cardiovascular Research), Partner Site Berlin, Berlin, Germany; 6 Dept. of Cardiothoracic Surgery, Charité–Universitätsmedizin Berlin, corporate member of Freie Universität Berlin, Humboldt-Universität zu Berlin, and Berlin Institute of Health, Berlin, Germany; Universite de Liege (B34), BELGIUM

## Abstract

The diagnosis of infective endocarditis (IE) remains a challenge. One of the rare bacterial species recently associated with biofilms and negative cultures in infective endocarditis is *Aerococcus urinae*. Whether the low number of reported cases might be due to lack of awareness and misidentification, mainly as streptococci, is currently being discussed. To verify the relevance and biofilm potential of *Aerococcus* in endocarditis, we used fluorescence *in situ* hybridization to visualize the microorganisms within the heart valve tissue. We designed and optimized a specific FISH probe (AURI) for *in situ* visualization and identification of *A*. *urinae* in sections of heart valves from two IE patients whose 16S rRNA gene sequencing had deteced *A*. *urinae*. Both patients had a history of urinary tract infections. FISH visualized impressive *in vivo* grown biofilms in IE, thus confirming the potential of *A*. *urinae* as a biofilm pathogen. In both cases, FISH/PCR was the only method to unequivocally identify *A*. *urinae* as the only causative pathogen for IE. The specific FISH assay for *A*. *urinae* is now available for further application in research and diagnostics. *A*. *urinae* should be considered in endocarditis patients with a history of urinary tract infections. These findings support the biofilm potential of *A*. *urinae* as a virulence factor and are meant to raise the awareness of this pathogen.

## Introduction

Infective endocarditis (IE) is a life-threatening infection of the endocardium and is associated with high morbidity and mortality [1]. Culture-negative endocarditis, where the causative pathogens remain elusive in the routine microbiological diagnostic work-up, represents a serious risk for the patient. Any antibiotic therapy must remain empirical and unadjusted to the microbial species or resistance patterns, which is associated with increased mortality [2]. Culture-negative results in routine microbiology diagnostics may be caused by biofilm growth of bacteria, prior antibiotic treatment of the patient, or by fastidious or slow-growing bacteria [3]. In recent years molecular biological methods such as nucleic acid amplification techniques (NAT) and fluorescence *in situ* hybridization (FISH) have helped detect microorganisms that might otherwise be missed by routine culture methods [4]. The direct detection of microorganisms in valve specimens by FISH enables the simultaneous visualization and identification of the causative agent *in situ*, also in culture-negative cases [5]. FISH provides information not only about the identity of the pathogen(s) present but also about their state of activity and spatial organization as planktonic or biofilm microorganisms. One of the rare bacterial species recently associated with culture-negative IE is *Aerococcus urinae* (*A urinae*) [6–10].

The genus *Aerococcus* was initially described in 1953 [11] and was first associated with IE in 1976 [12]. Subsequently, an *Aerococcus*-like organism emerged as a new urinary tract pathogen that caused local or systemic urinary tract infections (UTI) [13] and was involved in fatal endocarditis [14]. In 1992, this species was described as *A*. *urinae* [15]. Furthermore, there have been at least four additional *Aerococcus* species described in human infections, with *A*. *urinae* remaining the most relevant pathogen: *A*. *christensii* [16], *A*. *sanguicola* [17], *A*. *viridans* [18], and *A*. *urinaehominis* [19], which are involved in UTI, chorioamnionitis, endocarditis, and pneumonia [20, 21].

*A*. *urinae* is a Gram-positive coccus that produces alpha-hemolytic colonies on blood agar and is catalase-negative, and positive for leucine aminopeptidase. *A*. *urinae* may be misreported as alpha-hemolytic streptococci because of their similar growth characteristics, biochemical characteristics, and hemolysis pattern [8, 9]. Gram staining may further raise this suspicion, since streptococci appear as chains or pairs, whereas *A*. *urinae* typically appears in clusters or tetrads. Differentiation between these two genera is possible by hippurate hydrolysis testing or sequencing of the 16S rRNA gene [5, 21, 22]. Since 2013, MALDI-TOF (matrix-assisted laser desorption ionization time-of-flight mass spectrometry) has also played a major role in the identification from clinical samples of *A*. *urinae* [23, 24].

Concerning the treatment of *A*. *urinae* endocarditis, a combination of penicillin plus an aminoglycoside is recommended due to a described synergistic effect of the two drugs [21, 25, 26].

IE may be associated with microbial biofilms on the heart valves–microorganisms in sessile communities, which are more tolerant towards antibiotic treatment than their free-living, planktonic counterparts [27]. *A*. *urinae* isolated from human blood was shown *in vitro* to form biofilms and to trigger human platelet activation and aggregation. This may explain the potential virulence mechanisms of this pathogen, which may contribute to the ability of *A*. *urinae* to cause IE [6].

There are no direct data thus far on whether biofilm formation of *A*. *urinae* is relevant *in vivo*.

To further elucidate the biofilm formation of *A*. *urinae in vivo* we designed and optimized a species-specific FISH probe (AURI) to visualize and identify the pathogen in heart valves sections from two IE patients.

## Methods

### Specimen processing and fluorescence *in situ* hybridization (FISH)

Heart valve specimens from two patients were obtained during surgery at Charité–Universitätsmedizin and sent for FISH/PCR according to our standard routine diagnostics.

The tissue specimens were fixed intraoperatively with 3.7% formaldehyde in phosphate-buffered saline (pH 7.4) containing 50% ethanol at 4°C for 24 h, subsequently embedded in cold polymerizing resin and sectioned as previously described [28]. For hybridization, a pre-heated hybridization solution (20 μL) was mixed with 5 pmol of the respective oligonucleotide probe and the nucleic-acid-specific stain 4',6-diamidino-2-phenylindole (DAPI) and carefully applied to the tissue sections. Probes were 5´-end-labeled with either fluorescein 5-isothiocyanate (FITC) (EUB338) or indocarbocyanine Cy3 (AURI) and Cy5 (NONEUB338) (Biomers, Ulm, Germany).

After incubation for 2 h in a dark humid chamber at 50°C, the slides were rinsed with water, air-dried, and mounted with a mounting medium to prevent fading of the fluorescence signal (Vectashield, Vector Laboratories, California, USA).

For microscopy, an epifluorescence microscope (Axioplan2; Carl Zeiss, Jena, Germany) equipped with narrow-band filter sets (AHF-Analysentechnik, Tübingen, Germany) was used.

The analysis was performed in accordance with the ethical guidelines of the 1964 Declaration of Helsinki and approved by the Ethics committee of the Charité–Universitätsmedizin Berlin (E82/018/18). Since the study was performed as part of the routine diagnostic work-up, the need for informed consent was waived as per the ethics committee approval.

### Oligonucleotide probes

The probe EUB338 (5´-GCTGCCTCCCGTAGGAGT-3´) [29], which is complementary to a section of the 16S rRNA gene conserved in most microorganisms of the domain bacteria, was used to screen for bacterial colonization.

The probe NONEUB338, which is the antisense probe of EUB338, was used to exclude unspecific probe binding. In addition, a panel of FISH-probes for the detection of the species most prevalent in IE was applied as described [5]. To specifically detect *A*. *urinae*, we designed and optimized a 16S rRNA-directed FISH probe (AURI) (5´-AAGGCCATCGATAAGTGACAGCAA-3´). We compared the sequence of AURI with those of all 16S rRNA entries in the EMBL and GenBank databases to assess its specificity, and found 100% similarity only with the *A*. *urinae* 16S rRNA gene (November 2019).

### Bacterial strains

An *A*. *urinae* blood culture isolate from a patient not related to the cases presented here served as a positive control. *A*. *sanguinicola* (DSM 15633), the nearest phylogenetic neighbor with three mismatches in the probe binding site, was used as a negative control to optimize specific FISH conditions for AURI. Bacterial strains for the positive and negative controls were fixed as described elsewhere [30] and were included in each FISH experiment to control the probe specificity and sensitivity. In addition, we screened different Gram-positive cocci frequently associated with IE, all of which contained more than four mismatches at the probe-binding site of AURI (*Staphylococcus aureus* ATCC 25923, *S*. *epidermidis* PIA 8400 clinical isolate, *Streptococcus pyogenes* ATCC 19615, *Streptococcus pneumoniae* ATCC 6303; *Enterococcus faecalis* ATCC 29212, *E*. *faecium* ATCC 19434). The identity of all strains was confirmed by 16S rRNA gene sequencing.

### DNA processing and sequencing

DNA was extracted from sections of the embedded heart valve tissues and was then submitted to pan-bacterial amplification of part of the 16S rRNA gene using the primers TPU1 and RTU3, as previously described [28]. Amplicons were sequenced using a commercial sequencing facility (LGC Genomics, Berlin, Germany) and compared with all currently available sequences from the public databases (EMBL and GenBank) using BLAST and FastA of the sequence analysis program Husar 4.1 (Deutsches Krebsforschungszentrum, Heidelberg, Germany) and the commercial SmartGene program (SmartGene, Lausanne, Switzerland).

### Patient description

We analyzed heart valve samples from two IE patients. The first patient, a 67-year-old man, was admitted to the Charité–Universitätsmedizin Berlin with the diagnosis of acute dyspnea and suspected urosepsis. The anamnesis revealed that the patient had undergone empiric ciprofloxacin antibiotic treatment in his nursing home two months before due to acute cystitis. During this treatment, a urinary catheter was placed.

On admission, a systolic murmur compatible with mitral insufficiency was noted. Because of purulence in the urinary catheter and suspected urosepsis, empiric meropenem and ciprofloxacin antibiotic therapy was initiated immediately. A urine culture revealed growth of *Escherichia coli*.

The day after admission, a multiplane transesophageal echocardiogram (TEE) showed severe mitral insufficiency and a vegetation measuring 26 mm x 10 mm on the posterior leaflet, and the antibiotic therapy was escalated to vancomycin and gentamicin in addition to meropenem and ciprofloxacin. Because of his clinical deterioration, the patient underwent immediate mitral valve replacement surgery.

Two sets of blood cultures taken on the day before the surgery showed *Staphylococcus epidermidis* after one day of incubation. A third set of blood cultures taken on the day of the surgery revealed growth of *A*. *urinae* after two days of incubation. On the day of the actual surgery, three additional blood culture sets were taken, all of which again exhibited growth of *A*. *urinae*. The antibiogram of *A*. *urinae* from the blood cultures showed susceptibility to penicillin with an MIC of 0.004 mg/L. Antibiotic therapy was tailored to ampicillin and gentamicin.

A conventional routine diagnostic microbiology of the heart valve performed one week post-operatively confirmed *A*. *urinae* with susceptibility to ampicillin and penicillin (MIC values of 0.032 mg/L and 0.004 mg/L, respectively). After two weeks, treatment was switched to monotherapy with ampicillin. The postoperative course was uneventful.

The second patient, an 86-year-old woman, was transferred to Charité–Universitätsmedizin Berlin for aortic valve replacement after TEE in a geriatric hospital had revealed left ventricular enlargement and severe aortic insufficiency with a vegetation measuring 20 mm x 10 mm on the non-coronary cusp. In addition, she had been diagnosed with a UTI due to *Enterobacter cloacae*. Calculated antibiotic therapy with ampicillin, gentamicin and ceftriaxone was initiated four days before the surgery and tailored to ampicillin and gentamicin one day before the surgery after four sets of blood cultures indicated alpha-hemolytic streptococci. Aortic valve specimens were sent for conventional routine diagnostic microbiology, which showed Gram-positive cocci in the microscopic analysis but no growth in the culture.

Four days after the surgery, the bacteria from the preoperative blood cultures were identified as *A*. *urinae* and therapy was switched to penicillin and gentamicin for two days and was then further tailored to monotherapy with penicillin. The strain was susceptible to penicillin and cefotaxime (MIC values of 0.004 mg/L for both antibiotics) and resistant to gentamicin (MIC of 12 mg/L). The patient had an uneventful postoperative course.

## Results

Both heart valves were embedded and analyzed by FISH using the pan-bacterial probe EUB338 in order to screen for bacterial colonization including yet uncultivated or fastidious bacterial species. We found extensive areas with bacterial colonization and formation of microorganisms resembling structured biofilms, which were in part FISH-positive. None of the specific FISH probes for the species most commonly associated with IE identified the EUB338-positive bacteria. Therefore, *Staphylococcus* spp., *Streptococcus* spp., *Enterococcus* spp., as well as *Granulicatella*, *Tropheryma whipplei*, and *Bartonella* were excluded as infectious agents.

DNA was isolated from consecutive sections of embedded tissue and submitted to pan-bacterial PCR amplification. Sequencing of the PCR products resulted for the first patient in an 847-base pair fragment with the highest homology (99.6% identity) to the *A*. *urinae* 16S rRNA gene (accession number U64458). Similarly, for the second patient a 465-base pair fragment was obtained with the highest homology (100%) to the *A urinae* 16S rRNA gene (accession number CP002512).

**Fig 1:**
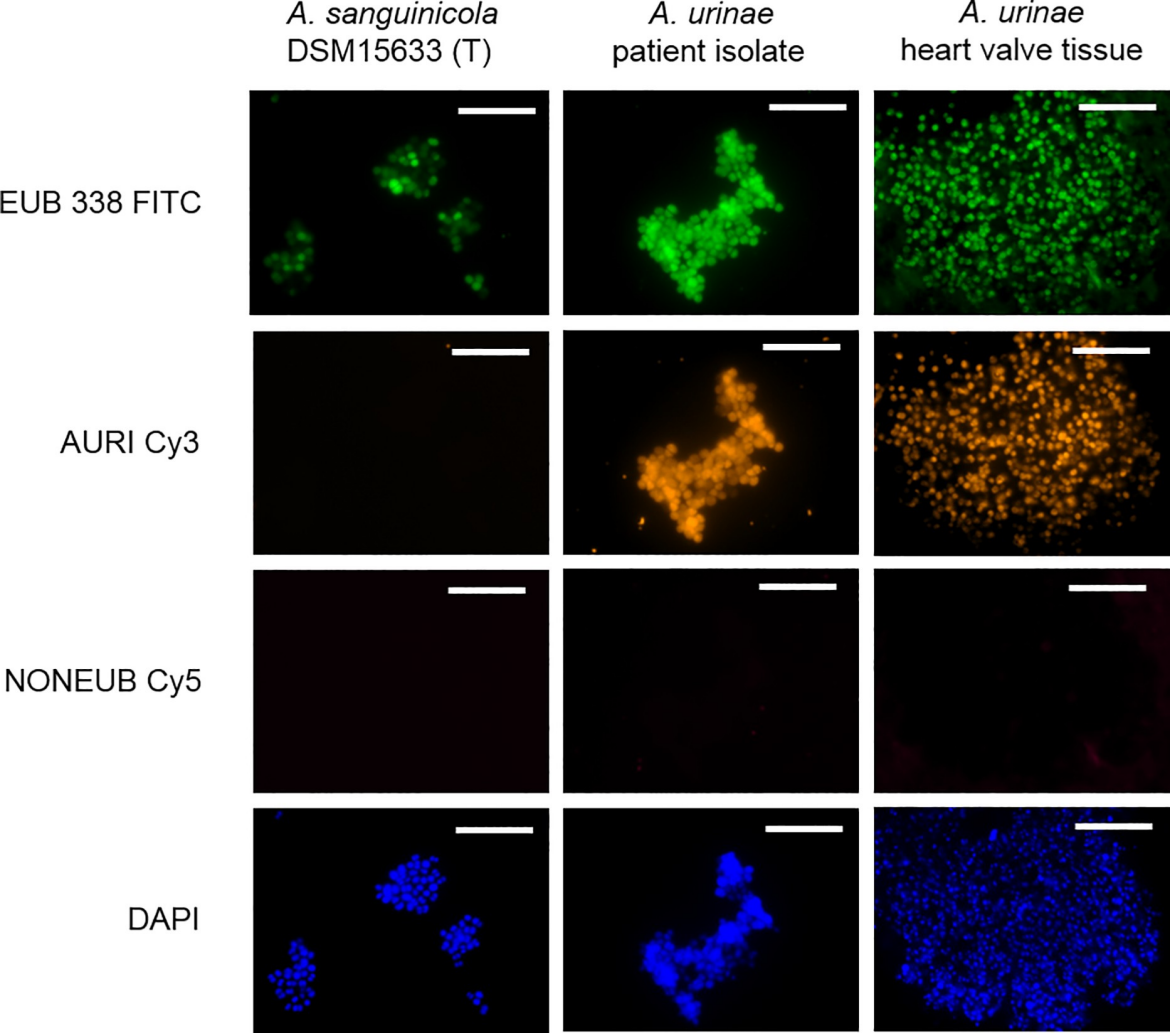
Specific identification of *Aerococcus urinae* by FISH. Fixed cultures of *Aerococcus sanguinicola* and *A*. *urinae*, as well as heart valve tissue were simultaneously hybridized with the probes EUB338 (FITC), AURI (Cy3) and NONEUB (Cy5). DAPI was used to stain nucleic acids. Each row shows an identical microscopic field with filter sets for FITC, Cy3, Cy5 and DAPI, respectively. AURI only detected *A*. *urinae* in the culture and the heart valve section, thus demonstrating specific hybridization. Scale bar 10 μm. Subsequently, the novel FISH probe AURI (Cy3-labeled) was applied to new sections of the heart valve samples simultaneously with the EUB338 (FITC-labeled); thus confirming *A*. *urinae* as the infectious agent of the IE *in situ* and demonstrating the biofilm potential of these bacteria (Figs 2 and 3).

For direct *in situ* identification of the microorganisms, an AURI FISH probe specific for *A*. *urinae* was designed. The specificity and sensitivity of the probe were evaluated *in silico* against all sequences available in the databases. The *in vitro* specificity and sensitivity were evaluated using a clinical isolate strain of *A*. *urinae* (positive control) and the nearest phylogenetic neighbor *A*. *sanguinicola* (three mismatches at the probe binding site, negative control). In addition, no false positive signal with AURI was obtained from a panel of Gram-positive cocci frequently associated with IE. The stringency of probe binding was adjusted by raising the formamide concentration in the hybridization buffer. AURI showed specific and strong hybridization signals at a formamide concentration in the range of 10-40% (v/v), clearly discriminating *A*. *urinae* from all other species tested (Fig 1).

## Discussion

The chances of survival of a patient in the ICU setting are 2.5 times higher when the correct antibiotic therapy is initiated as compared to empiric, broad-spectrum antibiotic therapy [2]. This makes species identification of the infectious agent highly desirable; however, this is not always successful. Reasons for culture-negative results may include biofilm growth, previous antibiotic treatment and/or rare or slow-growing bacterial species that are missed by routine culture methods.

We analyzed heart valve samples from two IE patients in whom routine culture methods did not immediately correctly identify the key causative pathogen.

In the first patient, culture revealed the presence of other bacteria such as *S*. *epidermidis* in the blood culture and *E*. *coli* in a urine sample. In addition, later on, a blood culture yielded *A*. *urinae*. In this case, PCR immediately identified *A*. *urinae* from the valve tissue and FISH confirmed *A*. *urinae* as the only causative agent (monospecies infection), revealing extensive structures consistent with microbial biofilms.

In the second case, *E*. *cloacae* was detected in the urine and the presence of alpha-hemolytic streptococci were suspected in blood culture. A blood culture later confirmed *A*. *urinae*. Again, FISH and PCR of the heart valve tissue immediately correctly identified the causative agent of the IE as *A*. *urinae*. Culture of the heart valve remained negative.

In both cases, FISH/PCR was the only method to unequivocally identify *A*. *urinae* as the only causative pathogen of IE, since all bacteria detected by the pan-bacterial probe were also positive with the specific AURI probe.

With the newly developed fluorescently labeled probe AURI specific *for A*. *urinae* described in this paper, we now have at our disposal a test system for further research and diagnostics for culture-negative heart valves that enables rapid and specific species identification and targeted antibiotic therapy.

Shannon et al. showed *in vitro* that blood culture isolates of *A*. *urinae* from IE patients form biofilms [6].

**Fig 2:**
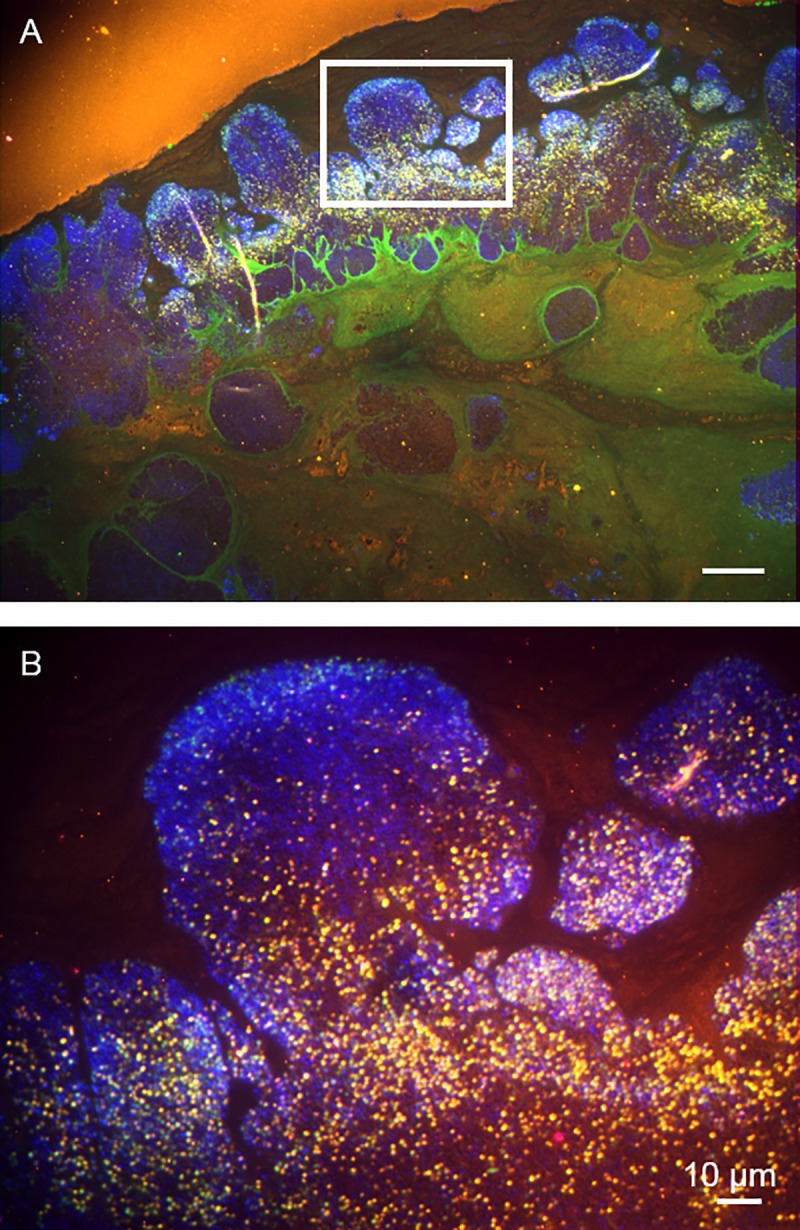
FISH reveals impressive biofilms on the mitral heart valve from the first patient, identifying the infectious agent as *Aerococcus urinae*. FISH on sections of the mitral valve tissue simultaneously using the probes AURI (Cy3-labeled), EUB338 (FITC-labeled), NONEUB (Cy5), and the nucleic acid stain DAPI (blue) identified the infectious agent *in situ* as *A*. *urinae*. We found impressive monospecies bacterial biofilms that were partly FISH-positive (yellow) and partly visible only with the nucleic acid stain DAPI (blue). The FISH signal corresponds to a high ribosome content and indicates bacterial activity at the time of surgery. A. Overview of the mitral valve tissue specimen showing impressive structures consistent with bacterial biofilms on the surface of the heart valve. B. Magnification of the inset marked in A shows distinct cells positive for the probes EUB338 and AURI, thus identifying the infectious agent *in situ* as *A*. *urinae*.

**Fig 3:**
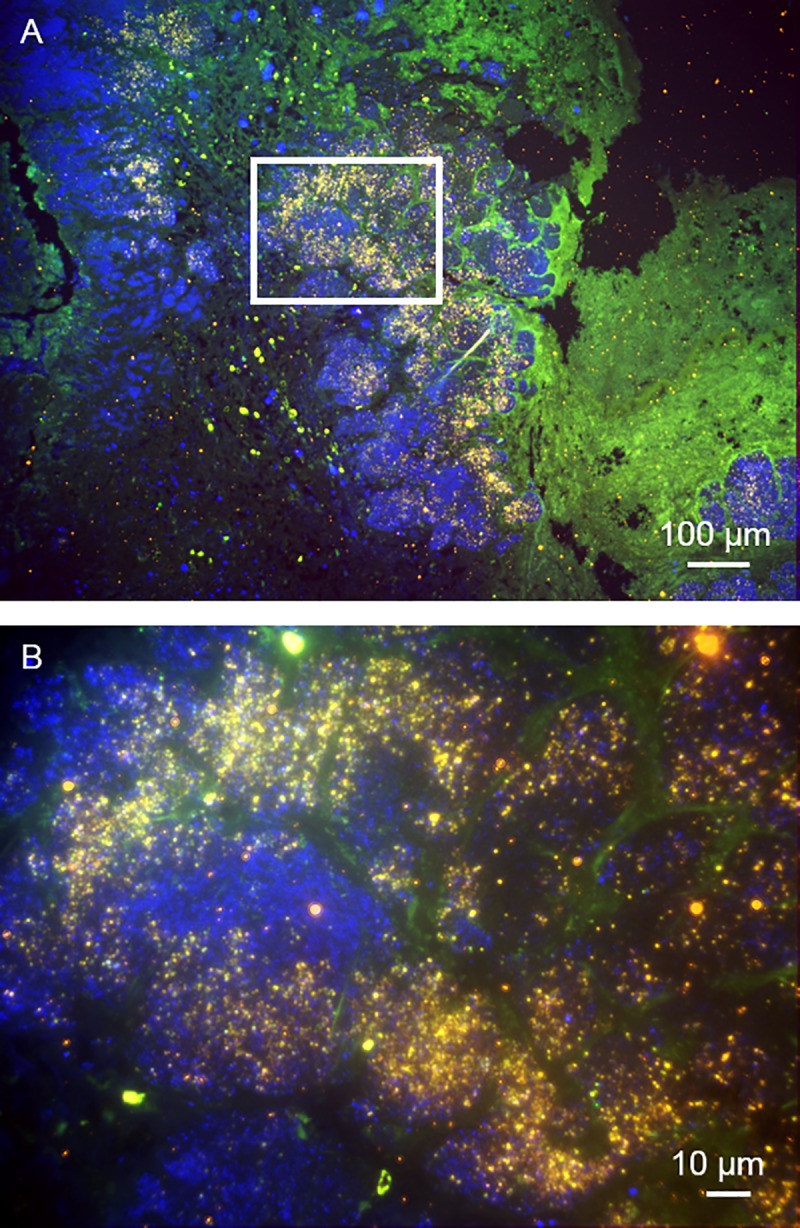
FISH reveals *Aerococcus urinae* infiltrating the aortic heart valve from the second patient. FISH of the aortic valve using the universal probe EUB338 (FITC), AURI (Cy3), NONEUB (Cy5), and the nucleic acid stain DAPI identifies *A*. *urinae in situ*. A. Overview of the aortic valve tissue specimen displaying large areas consistent with bacterial biofilms. (B) Magnification of the inset marked in A shows FISH-positive cells for the probes EUB338 and AURI (yellow) as well as DAPI-only positive cells (blue) within the heart valve tissue. Note the destructive growth of *A*. *urinae* in the heart valve tissue.

Here we show, to our knowledge for the first time, that impressive communities of *A*. *urinae* also exist in *ex vivo* material. A biofilm is determined by the arrangement of bacterial communities and the matrix in which they are embedded. While this matrix is composed of only bacterial products in vitro, in biofilm-associated infections it is also formed by the host's own substances such as fibrin, blood components, and tissue components [27]. Although FISH is unable to analyze the matrix in which the bacteria are located, it is still able to visualize the typical biofilm property of structural organization of the bacteria: the fluorescence signal intensity of FISH correlates with the bacterial ribosome content and therefore demonstrates activity of the bacteria [31, 32]. The cases presented here display areas with a higher ribosome content correlated to more active areas, which indicates a spatial organization of the bacteria in the so-called mushroom formations (Fig 2 and to a lesser extent Fig 3).

Therefore, these images show *in vivo* grown *A*. *urinae* biofilms *in situ* in IE and confirm *A*. *urinae* as the biofilm pathogen. This is a relevant finding, as bacteria grown as a biofilm exhibit a greater recalcitrance to antibiotic treatment than free-living, planktonic microorganisms [27]. This might be particularly important for beta-lactam antibiotics, which act on cell wall synthesis and therefore are not as active on dormant cells which might rest in the less active parts of the biofilms. This hypothesis supports the data of Skov et al. (2001) who recommend the addition of gentamicin in IE based on time-kill curves *in vitro* [25]. Therefore, we think that diagnostic biofilm staging (Specific clinical guidelines for the antibiotic treatment of biofilm-associated infections, however, still need to be further developed [27]. planktonic cells, microcolonies, nascent or mature biofilms) is necessary to correlate therapeutic efficacy to the biofilm state and consequently to improve antibiotic algorithms.

## Limitations

Unfortunately, this study is limited to a mere two cases; however, we hope to analyze more cases now that the FISH-probe is ready available. One limitation of FISH as a diagnostic tool is its application in the post-surgical setting only. Awareness of thorough documentation of the patient history including UTI must be raised, and respective sampling must be initiated in order to diagnose a possible *Aerococcus* involvement more timely.

## Conclusions

In conclusion, *A*. *urinae* should be considered in IE patients with a history of UTI. Clearly, the biofilm potency of *A*. *urinae* as a virulence factor must be taken into account and awareness of *A*. *urinae* as a relevant pathogen in UTI and its association with IE must be raised.
